# Implementing Age Friendly Health Systems: Enhancing Care for Older Veterans

**DOI:** 10.1093/geroni/igaf122.3165

**Published:** 2025-12-31

**Authors:** Lana Brown, Hallie Keller, Olive Phillips

**Affiliations:** Central Arkansas Veterans Healthcare System, North Little Rock, Arkansas, United States; Central Arkansas Veterans Healthcare System, North Little Rock, Arkansas, United States; Central Arkansas Veterans Healthcare System, North Little Rock, Arkansas, United States

## Abstract

The Age Friendly Health Systems (AFHS) is an initiative of The John A. Hartford Foundation and the Institute for Healthcare Improvement, in partnership with the American Hospital Association and the Catholic Health Association of the United States. In March 2020, the Veterans Affairs’ (VA) Office of Geriatrics and Extended Care joined the AFHS movement to deliver safe, reliable, high-quality, evidence-based health care in every setting based on what matters most to older Veterans and their caregivers. AFHS entails providing four evidence-based elements of high-quality care, known as the “4Ms,” to older adults: What Matters, Medication, Mentation, and Mobility. One VA Geriatric Patient-Aligned Care Team received AFHS Level II recognition in January 2023. Forty-two Veterans, 6 health care providers (MDs/APRNs), and 7 nurses were surveyed in 2024 to gather opinions regarding Age Friendly care. Four out of 6 providers agreed that AFHS practices were worthwhile to improve care of Veterans, and none expressed difficulty aligning “What Matters” to the Veteran’s plan of care. Five out of 7 nurses reported that assisting Veterans with AFHS duties was not burdensome, and none found it difficult to complete screening. Veterans agreed that they felt respected and comfortable during their visit (95%), providers listened to them carefully (98%), providers included what matters most in plans for managing health and wellbeing (95%), and providers talked with them about specific goals for health (92%). Staff and Veteran responses suggest that AFHS practices were successfully adopted, fostering plans of care that align with Veteran goals and preferences.

